# Serotype distribution and antimicrobial resistance of *Streptococcus pneumoniae* causing invasive diseases in China: a meta-analysis

**DOI:** 10.1186/s12887-019-1722-1

**Published:** 2019-11-11

**Authors:** Jinjian Fu, Rongsong Yi, Yongjiang Jiang, Shaolin Xu, Peixu Qin, Zhuoxin Liang, Jichang Chen

**Affiliations:** 1 Department of Laboratory Medicine, Liuzhou Maternity and Child Health Care Hospital, Liuzhou, 545001 Guangxi China; 2 Department of Laboratory Medicine, Affiliated Rong’an of Liuzhou Maternity and Child Health Care Hospital, Liuzhou, 545001 Guangxi China; 3 Department of Pediatric, Liuzhou Maternity and Child Health Care Hospital, Liuzhou, 545001 Guangxi China; 4 Department of Neonatology, Liuzhou Maternity and Child Health Care Hospital, Liuzhou, 545001 Guangxi China; 5 Department of Pediatric Intensive Care Unit, Liuzhou Maternity and Child Health Care Hospital, Liuzhou, 545001 Guangxi China; 6 Department of Pediatric, Affiliated Rong’an of Liuzhou Maternity and Child Health Care Hospital, Liuzhou, 545001 Guangxi China

**Keywords:** Invasive pneumococcal disease, Children, China, Serotype, *Streptococcus pneumonia*

## Abstract

**Background:**

To summarize information about invasive pneumococcal disease (IPD) among children in mainland China.

**Methods:**

Sixteen eligible studies were included in this systematic review and the random effect model was used to estimate the pool prevalence of IPD.

**Results:**

The most predominant serotypes circulating in children were 19F (27.7, 95% confidence interval (95% CI): 17.7–37.6%), 19A (21.2%, 16.4–26.1%), 14 (16.5%, 12.8–20.1%), 6B (8.6%, 5.2–10.8%) and 23F (7.3%, 5.2–9.5%). The serotype coverage of the available pneumococcal conjugate vaccines PCV7, PCV10, and PCV13 was 60.8% (52.5–69.4%), 65.1% (57.7–72.4%), and 90.0% (87.1–92.8%), respectively. The pooled antibiotic resistance rates of *Streptococcus pneumoniae* revealed a resistance to penicillin prevalence rate of 32.0% (12.1–51.9%). Approximately 94.4% (90.7–98.1%) and 92.3% (87.4–97.3%) of isolates were resistant to erythromycin and clindamycin. eBURST analysis revealed great diversity among isolates, with 102 sequence types (STs) for 365 isolates. The major predominant clonal complexes (CCs) were CC271 (43.6%, 159/365), CC876 (13.4%, 49/365), CC81 (5.2%, 19/365), and CC90 (4.1%, 15/365). Long-term and regional surveillance of *S. pneumoniae* is necessary.

**Conclusions:**

Based on our pooled results showing that PCV13 coverage of the reported serotypes was 90% and that most serotypes contributed to the distribution of antibiotic-resistant isolates, implementation of PCV13 into the Chinese Expanded Program on Immunizations (EPI) would achieve health benefits in Chinese children.

## Background

*Streptococcus pneumonia* (*S. pneumoniae*) is one of the most prominent pathogens, causing mild to life-threatening invasive diseases due to its well-known capsule pathogenicity [1]. It has been reported that approximately 1 million people die of pneumococcal diseases annually, most of whom are children under 5 years old [2–4]. Pneumococcal conjugate vaccines (PCVs) targeting 7, 10, or 13 of more than 90 serotypes of *S. pneumoniae* have been successively introduced to reduce the burden of invasive pneumococcal disease (IPD) in vaccinated children in developed countries [5, 6]. In fact, it has been reported that after licensing PCV13, pneumococcal diseases of any cause, but especially those caused by PCV13 minus PCV7 serotypes, were further decreased across each age group in the UK [7].

In China, *S. pneumoniae* is one of the most common pathogens and can cause infectious diseases, especially pneumonia, in children under 5 years of age [8]. PCV7 was first introduced in mainland China in 2008 [9] but due to its high price was not included as part of the national immunization schedule. Because of its high cost in the the private market [10], and the vaccine may not be available to some low-income families, especially in western China. But a model predicted that every year, 112,629 cases of pneumococcal-related disease could be prevented in Shanghai if the PCV7 vaccine could be introduced [11]. After employing PCV7, another multicenter study conducted in Shanghai in 2013 reported that the serotype coverage of PCV7, − 10, and − 13 was 58.6, 59.4 and 85.1%, respectively [12].

Both genetic background and capsular type contribute to the ability of *S. pneumoniae* to cause invasive diseases [13, 14]. The aim of this study was to obtain a systematic estimate of the serotypes of *S. pneumonia* and their antibiotic resistances and to determine clonal types causing IPD in mainland China.

## Material and methods

### Literature search strategy

The design and construction of this systematic review was performed and completed according to the PRISMA checklist [13]. (see Additional file 1). The following databases were searched: PubMed, MEDLINE, EMBASE, WanFang Data (http://www.wanfangdara.com.cn), and the China National Knowledge Infrastructure (CNKI) database (http://cnki.net/). The time frame was January 2000 to September 2016. The keywords for the literature search were “*Streptococcus pneumoniae* OR invasive pneumococcal disease OR pneumococcal conjugate vaccine”, “children OR school children OR 0-18 years”, “serotype OR capsular type” and “China OR Chinese”.

### Eligibility criteria

Two reviewers (RS Yi and JC Chen) independently identified eligible studies and extracted data based on the following criteria: (1) the publication language was Chinese or English, (2) the study participants were generally Chinese children aged from 0 to 18 years, (3) the study provided invasive pneumococcal serotypes and/or specific patterns of antibiotic sensitivity, and (4) the serotyped pneumococcal isolates were obtained from normally sterile sites (e.g., blood, cerebrospinal fluid, and pleural fluid). Exclusion criteria were as follows: (1) the study data included adult patients, and specific infection data about children could not be extracted; (2) the data were not regarding invasive pneumococcal diseases; (3) the data did not provide specific serotypes and/or specific patterns of antibiotic sensitivity; (4) the studies were reviews, lectures, or editorials; or (5) the studies were duplicates published in both Chinese and English (in this case, only the English-language studies were included).

### Data extraction

The data were screened and reviewed independently by two authors (RS Yi and JC Chen) according to the inclusion and exclusion criteria, and the following information was extracted: first author’s name; study location; district; study period; publication year; age group; positive strains; serotyping method; serotypes; serotype coverage of PCV7, − 10, and − 13; and antibiotic resistance patterns of the positive cases and total cases.

### Statistical analysis

Statistical analyses were performed using Stata statistical software version 13.0 (Stata corporation LP, College Station, Texas, USA). The pooled relevant serotypes and antibiotic resistance prevalence and their corresponding 95% confidence intervals (CIs) were evaluated using the DerSimonian and Laird random-effects model [15]. The Cochrane chi-square (*x*^*2*^) test and quantification with the *I*^*2*^ statistic were used to calculate the source of heterogeneity [16, 17]. Begg’s funnel plots and Egger’s test (significant at *P* ≤ 0.1) were used to assess publication bias [18].

## Results

### Characteristics of the studies

**Fig. 1:**
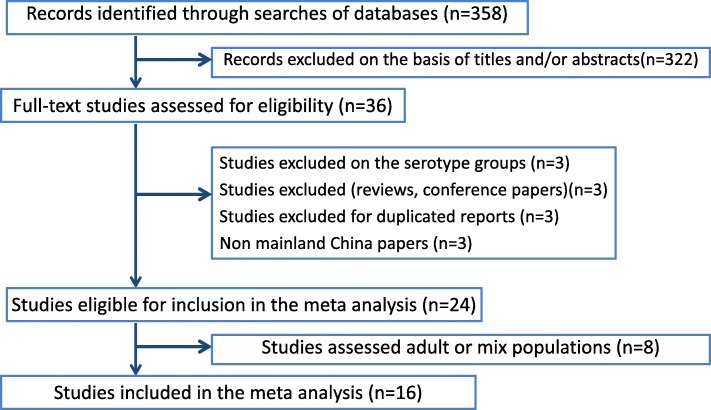
Flow chart of the study selection process

The literature search and study selection process in the final analysis is shown in Fig. 1. A total of 358 articles were identified. After screening the titles and abstracts and based on our inclusion/exclusion criteria, 24 studies were selected for further investigation. Studies involving adult or mixed populations were excluded, and 16 studies, with 2146 patients, were selected for the final meta-analysis [19–34].

The main characteristics of the studies are listed in Table 1. The first study of invasive pneumococcal diseases among children was conducted in 8 centers in 2005–2006 [30]. Only 3 investigations were conducted before PCV7 was introduced in mainland China [19, 21, 30]. The geographical characteristics of the included surveys revealed that 5 were conducted in northern China [21, 29, 31, 32, 34]and 8 in southern China [20, 22, 23, 25–28, 33]. All samples evaluated in the included studies were subjected to the Quellung reaction method, except for 4 samples for which the multiple polymerase chain reaction (MPCR) method was applied. The ages of the children investigated ranged from 0 to 14 years.

**Table 1:** Characteristic of included studies

Author	Study location	District	Study period	Publication year	Age group	Total strains	Positive strains	Serotyping method
Xue L et al. [19]	11 centers	national	2006–2008	2010	< 14 years	171	171	Quellung reaction
Ma X et al. [20]	Shenzhen	south	2009–2012	2013	< 14 years	87	87	Quellung reaction
Liu C et al. [21]	Shenyang	north	2009–2011	2013	< 5 years	61	61	Quellung reaction
Ding Y et al. [22]	Suzhou	south	2011–2013	2015	< 14 years	79	79	MPCR
Kang L et al. [23]	Chongqing	south	2010–2013	2016	< 11 years	51	51	MPCR
Liu C et al. [24]	15 centers	national	2005–2008	2010	< 11 years	148	81	Quellung reaction
Xu F et al. [25]	Nnajing	south	2007–2010	2012	< 14 years	48	48	Quellung reaction
Lu C et al. [26]	Shenzhen	south	2010–2013	2015	< 14 years	74	74	Quellung reaction
Zhou K et al. [27]	Nanjing	south	2009–2013	2015	< 14 years	51	51	Quellung reaction
Song X et al. [28]	Taizhou	south	2010–2014	2015	< 5 years	322	322	Quellung reaction
Wang Y et al. [29]	Hebei	north	2014	2016	< 8 years	43	43	MPCR
Lyu S et al. [30]	Beijing	north	2013–2014	2016	< 14 years	21	21	Quellung reaction
Liu Y et al. [31]	8 centers	national	2005–2006	2008	< 5 years	451	31	Quellung reaction
Dong F et al. [32]	Beijing	north	2013–2014	2016	< 14 years	195	21	Quellung reaction
Li J et al. [33]	Beijing	north	2012–2014	2016	< 14 years	21	21	Quellung reaction
Miao D et al. [34]	Nanjing	south	2007–2011	2016	< 14 years	323	56	MPCR

*MPCR* multiple polymerase chain reaction

### Serotypes of pneumococcal strains isolated from IPD pediatric patients

Tables 2 and 3 present the pooled serotype distributions of *S. pneumoniae* among Chinese children aged from 0 to 14 years and under 5 years, respectively.

**Table 2:** Pooled serotype distribution of *Streptococcus pneumoniae* among Chinese children

Variable	Included studies	Positive cases	Total cases	Rate (%) (95% CI)
Total IPD	16	1218	2146	10.0 (5.0–14.9)
1	3	9	155	4.9 (1.5–8.3)
3	1	4	56	7.1 (0.4–13.9)
4	1	1	61	1.6 (1.5–4.8)
5	6	13	368	2.6 (1.0–4.2)
6A	1	1	21	4.8 (4.3–13.9)
6B	3	96	1006	8.0 (5.2–10.8)
7F	3	8	173	4.5 (1.4–7.6)
9 V	7	23	357	5.7 (3.3–8.1)
14	11	140	796	16.5 (12.8–20.1)
18C	15	5	171	2.9 (0.45.4)
19A	15	241	1197	21.2 (16.4–26.1)
19F	13	419	1197	27.7 (17.7–37.6)
23F	14	94	1062	7.3 (5.2–9.5)
PCV7 serotype coverage	14	752	1197	60.8 (52.2–69.4)
PCV10 serotype coverage	14	784	1197	65.1 (57.7–72.4)
PCV13 serotype coverage	14	1030	1197	90.0 (87.1–92.8)

**Table 3:** Pooled serotype distribution of *Streptococcus pneumoniae* among Chinese children under 5 years old

Variable	Included studies	Positive cases	Total cases	Rate (%)(95% CI)
Total IPD	5	517	590	
1	1	2	61	3.3 (1.2–7.7)
4	1	1	61	1.6 (1.5–4.8)
5	2	5	67	7.0 (0.9–13.0)
6A	1	1	36	2.8 (2.6–8.1)
6B	3	53	523	7.8 (1.0–14.7)
9 V	2	4	97	3.8 (0.0–7.7)
14	4	49	268	15.1 (6.6–23.6)
19A	5	115	590	25.8 (15.5–36.0)
19F	5	242	590	26.4 (4.0–48.8)
23F	5	56	590	8.4 (5.5–11.2)
PCV7 serotype coverage	5	403	590	53.0 (32.7–73.3)
PCV10 serotype coverage	5	412	590	57.4 (39.7–75.2)
PCV13 serotype coverage	5	517	590	83.5 (72.8–94.1)

Sixteen studies [19–34] reported serotypes for invasive pneumococcal diseases. A total of 18 different serotypes were identified among 1218 strains, with the most predominant being 19F. The pooled prevalence of the 19F serotype was 27.7% (17.7–37.6%), followed by the 19A serotype at 21.2% (16.4–26.1%), the 14 serotype at 16.5% (12.8–20.1%), the 6B serotype at 8.0% (5.2–10.8%) and the 23F serotype at 7.3% (5.2–9.5%). Of the 1218 isolates, 752, 784, and 1030 were identified as serotypes included in the coverage of PCV7, − 10, and − 13, respectively. The serotype coverage rates were 60.8% (52.2–69.4%), 65.1% (57.7–72.4%), and 90.0% (87.1–92.8%) for PCV7, − 10, and − 13, respectively.

Only 5 studies [19, 21, 24, 28, 30] reported IPD strains from children under 5 years of age. A total of 13 different serotypes were identified among 517 strains, with 19F being the most predominant serotype. The pooled prevalence of the 19F serotype was 26.4% (4.0–48.8%), followed by the 19A serotype at 25.8% (15.5–36.0%), the 14 serotype at 15.1% (16.6–23.6%), the 23F serotype at 8.4% (5.5–11.2%) and the 6B serotype at 7.8% (1.0–14.7%). Of the 517 isolates, 403, 412, and 517 were identified from children under 5 years old as serotypes included in the coverage of PCV7, − 10, and − 13, respectively. The serotype coverage rates were 53.0% (32.7–73.3%), 57.4% (39.7–75.2%), and 83.5% (72.8–94.1%) for PCV7, − 10, and − 13, respectively. Significant heterogeneity (*P* values all < 0.001) was found for each serotype and the coverage of PCV7, − 10, − 13.

### Antibiotic resistance patterns of IPD isolates

Tables 4 and 5 show the antibiotic resistance patterns of IPD isolates among Chinese children. Ten studies reported antibiotic resistance patterns among the 0–14 year-old group. All of the isolates were highly resistant to erythromycin, clindamycin, tetracycline, and sulfamethoxazole, with resistance rates of 94.4% (90.7–98.1%), 92.3% (87.4–97.3%), 83.7% (75.1–92.2%), and 74.4% (64.5–84.4%), respectively. The reported penicillin-non-susceptible *S. pneumoniae* (PNSP) rate was 74.6% (23.3–71.9%), and the resistance rate to penicillin was 32.0% (12.1–51.9%).

**Table 4:** Pooled antibiotic resistance rates of *Streptococcus pneumoniae* among Chinese children

Antibiotics	Included studies	Positive cases	Total cases	Rate(%) (95% CI)
Penicillin	9	488	1345	32.0 (12.1–51.9)
Amoxicillin clavulanic acid	5	199	1026	16.1 (2.1–30.0)
Amoxicillin	2	61	159	37.5 (27.6–47.4)
Ceftriaxone	7	239	1216	14.7 (6.1–23.3)
Chloramphenicol	7	129	910	11.4 (7.2–15.6)
Erythromycin	10	1259	1396	94.4 (90.7–98.1)
Clindamycin	8	1066	1204	92.3 (87.4–97.3)
Sulfamethoxazole	10	1052	1396	74.4 (64.5–84.4)
Tetracycline	9	1098	1335	83.7 (75.1–92.2)
PNSP	10	693	1396	74.6 (23.3–71.9)

*PNSP* Penicillin-Non-susceptible *Streptococcus pneumonia*

**Table 5:** Pooled antibiotic resistance rates of *Streptococcus pneumoniae* among Chinese children under 5 years old

Antibiotics	Included studies	Positive cases	Total cases	Rate(95% CI)
Penicillin	3	407	834	45.1 (18.5–71.7)
Amoxicillin clavulanic acid	3	195	834	23.3 (20.4–26.2)
Ceftriaxone	3	211	834	22.5 (15.3–29.7)
Chloramphenicol	1	79	451	17.5 (14.0–21.0)
Erythromycin	3	713	834	87.8 (74.8–99.3)
Clindamycin	3	705	834	84.2 (71.2–97.1)
Sulfamethoxazole	3	660	834	83.9 (68.6–99.2)
Tetracycline	2	631	773	78.9 (45.6–99.3)
PNSP	3	520	834	60.0 (17.2–89.4)

*PNSP* Penicillin-Non-susceptible *Streptococcus pneumoniae*

The antibiotic resistance patterns of IPD in the under 5 years-old group were similar to those of the 0–14 years-old group. Predominant resistance was observed for erythromycin at 87.8% (74.8–99.3%), followed by clindamycin at 84.2% (71.2–97.1%), sulfamethoxazole at 83.9% (68.6–99.2%), and tetracycline at 78.9% (45.6–99.3%). The PNSP rate was 60.6% (17.2–89.4%) and the penicillin resistance rate 45.1% (18.5–71.7%). Significant heterogeneity (*P* values all < 0.001) was reported for each antibiotic resistance rate.

### Genetic background of IPD strains isolated from children in mainland China

A total of 102 STs were reported in the 365 IPD isolates for which multilocus sequence typing (MLST) was conducted. The five most highly predominant STs for all reported IPD isolates were ST271 (20.82%, 76/365), ST320 (19.73%, 72/365), ST876 (12.33%, 45/365), ST81 (3.84%, 14/365) and ST90 (3.56%, 13/365), which were mainly related to serotypes 19F, 14, 19A, and 23F. The five most predominant STs among the 102 reported accounted for 60.28% of the isolates.

**Fig. 2:**
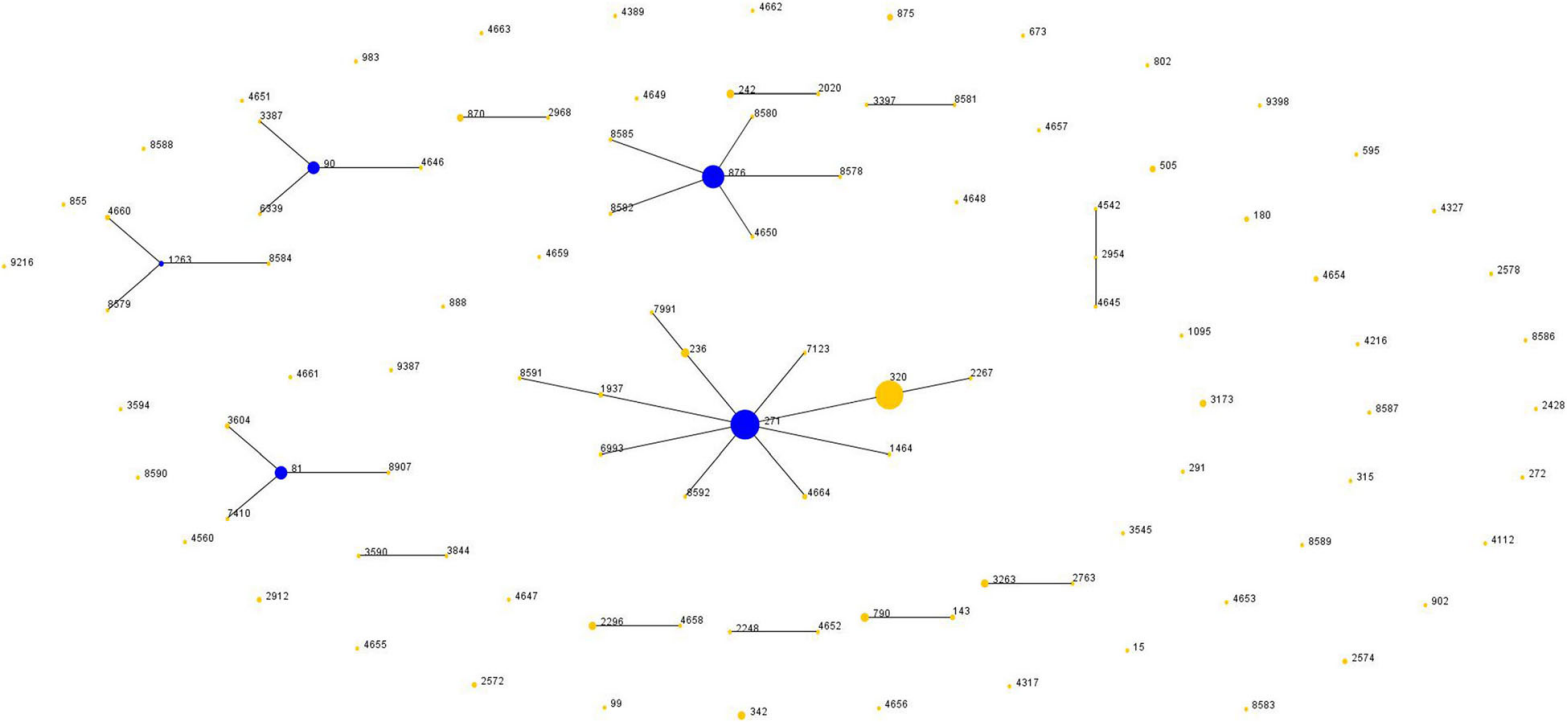
Population snapshot of pooled *Streptococcus pneumoniae* data based on eBURST analysis. One spot corresponds to the number of pneumococcal isolates with the same ST

**Fig. 3:**
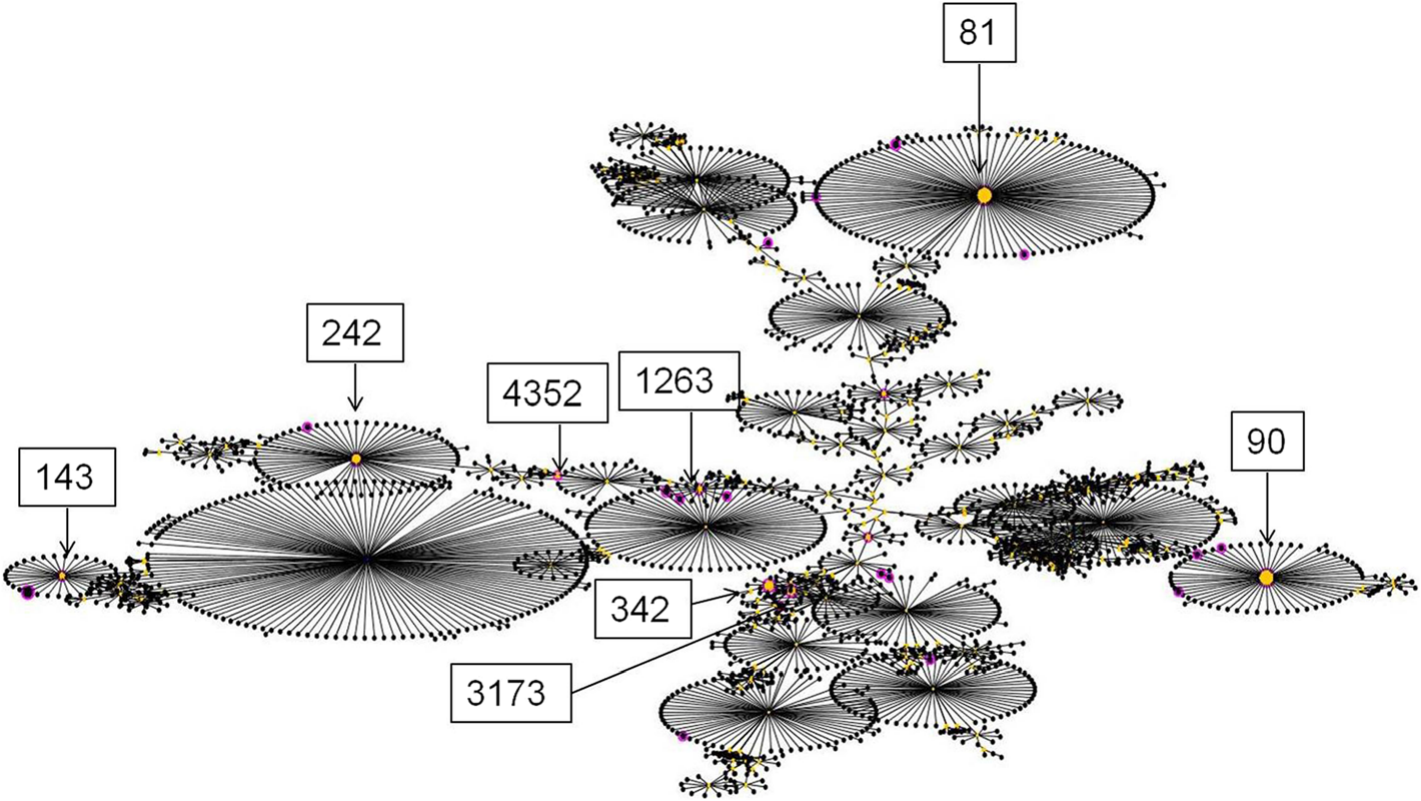
Pooled *Streptococcus pneumoniae* data based on global database screening using eBURST analysis. One spot corresponds to the number of pneumococcal isolates with the same ST. The yellow spot indicates the major clone found in our study. The pink spot indicates clones found in our study

MLST data analysis performed using eBURST v3 grouped the isolates into 4 CCs. ST81, ST90, ST876 and ST271 were confirmed as probable founder genotypes (Fig. 2). Eleven single-locus variants (SLVs) were found in ST271. Figure 3 shows high genetic diversity of IPD isolates from mainland China based on the global *S. pneumoniae* genetic background.

## Discussion

Epidemiological and clinical studies have shown that invasive pneumococcal diseases caused by *S. pneumoniae* is a major public health burden. Based on a systematic review of the literature, Chen et al [35] reported that approximately 700,000 children are diagnosed with pneumococcal disease each year and that approximately 30,000 deaths are attributed to *S. pneumoniae*; moreover, over 50% of children with pneumonia in China died because of this pathogen. The high pneumococcal disease burden makes the investigation and evaluation of *S. pneumoniae* serotypes important for providing evidence and guidance for the use of vaccines and antibiotics targeting the pathogen.

Our study revealed that the predominant serotypes circulating among Chinese children were 19F, 19A, 14, 6B and 23F. For children under 5 years old, the predominant serotypes were 19F, 19A, 14, 23F and 6B. A global serotype project confirmed that serotype 14 was the most prevalent cause of IPD among children under five in every region, including Asia; however, this systematic review did not include studies from mainland China, which contains the largest population of children under 5 years of age in the world [36]. In that report, seven serotypes (1, 5, 6A, 6B, 14, 19F and 23F) accounted for over 50% of IPD in every region, including Asia, and our results demonstrated that 14, 19F, 6B, 23F and 19A accounted for 80.7 and 73.5% of all IPD in Chinese children 0–14 years and children under 5 years of age, respectively. The major serotype patterns of *S. pneumoniae* in mainland China were consistent with the ANSORP study, which revealed that 19F, 23F, 19A, 14 and 6B were the major serotypes in Asia [37]. Serotype 1 is reported as one of the 5 most common serotypes in many developing countries such as in South Africa Kenya and in Philippines [37–39], but this serotype was infrequency in our study, which may revealed that the serotype distribution of S.pneumoniae was varied geographically [40].

Pooled analysis of multiple surveillance sites revealed that vaccine-serotype and overall IPD rates declined consistently and significantly after PCV7 introduction, and this effect was still observed after 7 years [41]. Unfortunately, these promising effects may not represent the important public health implications in some countries that did not implement routine immunization with PCVs, such as China [10]. Although the PCV7 vaccine was imported into and licensed in China in 2008, this vaccine has not yet been included in the Chinese Expanded Program on Immunizations (EPI) [42]. The PCV7 vaccine was removed from the market in 2015, though new vaccines, such as PCV10 and PCV13, have not yet been employed in the Chinese market, making the situation even worse [10].

Our analyses revealed serotype coverage rates for PCV7, − 10, and − 13 of 60.8, 65.1, and 90.0%, respectively, for IPD among Chinese children. A cohort and case-control study conducted in Europe [43] revealed that after the 13-valent pneumococcal conjugate vaccination was introduced, the overall IPD incidence declined in both vaccinated (hazard ratio, HR: 0.24, 95% CI: 0.14–0.40) and unvaccinated children (HR, 95% CI: 0.22, 0.09–0.55). The direct effect of PCV13 against vaccine serotypes was as high as 95%. According to two studies conducted in China, only 9.9% or 10.1% of children received a dose of PCV7 [10, 44]. Such low vaccination may not produce satisfactory results for reducing the burden of pneumococcal diseases. A Markov simulation model conducted by Mo et al [45] showed that PCV13 can reduce IPD by 31.3% and pneumonia by 15.3% in mainland China.

The high pneumococcal disease burden and the largest population baseline of children in China means that the cost-effectiveness of launching PCV7 or higher valence vaccines would improve if the government introduced the vaccines into the EPI. According to the mathematical model conducted by Hu et al [46], a policy of universal PCV7 vaccination among infants in China would prevent approximately 10.8 million cases of disease and save 636,371 lives over 10 years, largely due to the indirect effectiveness of the vaccine on the unvaccinated population.

Global multi-region studies have revealed that the highest antibiotic resistance to *S. pneumoniae* was consistently found in Asia [37], and the ANSORP reports demonstrated that *S. pneumoniae* isolates from mainland China had the highest antibiotic resistance rates [37]. Indeed, it was reported that *S. pneumoniae* isolated from Chinese children had the highest antibiotic resistance rates [31]; the most predominant resistance, 96.4%, was to the antibiotic erythromycin, followed by tetracycline and sulfamethoxazole. Our study revealed *S. pneumoniae* to be most frequently resistant to erythromycin, followed by clindamycin, tetracycline, and sulfamethoxazole, consistent with previous study and the ANSORP study [37].

Our findings indicated a pooled penicillin resistance rate to *S. pneumoniae* of 32.0% and a PNSP of 74.6%, which was much higher than the ANSORP study results but consistent with a study conducted in Beijing that revealed a total non-susceptibility rate of penicillin of 91.5% if based on an oral breakpoint of *Clinical and Laboratory Standards Institute* (CLSI) [31]. The limitation of our study is that we cannot estimate the resistance rate to penicillin based on revised CLSI breakpoints, as most of the included studies did not report the adaptation of the breakpoints. However, our results were partially consistent with the ANSORP study, which reported a resistance rate to penicillin of 60.0% for meningeal isolates [37].

The snapshot from eBURST showed a great diversity among IPD strains isolated in mainland China. The predominant clones were CC271, CC876, CC81 and CC90. CC271 was the most predominant CC, and the inclusion of 11 STs, such as ST271, ST320, ST236, and ST4664, accounted for 43.6% of all the included isolates from the literature. Several studies have confirmed that this clone became prevalent prior to the introduction of PCV7 and spread rapidly among both adults and children in China [44]. The widespread use of antibiotics has also been implicated in the emergence of serotype 19A and 19F isolates in both communities and hospitals. The two serotypes of the isolates were reported to be multidrug resistant and carriers of the *ermB* and *mefA* genes, making the situation even worse [47, 48].

A significantly increased risk of CC271 strains, which caused IPD in our study, is anticipated among children and is in agreement with existing trends in the literature regarding transmission [49]. ST236, namely the original Taiwan^19F^-14 clone, was the major international antibiotic-resistant strain. In addition, long-term monitoring data revealed that ST271 and ST320 evolved rapidly and replaced ST236 due to their higher fitness and that they were becoming well established in local regions of China [47]. It was also reported that CC271 is prevalent in Asian countries, such as Japan and Korea, due to the introduction of PCV7 [50, 51]. After implementation of PCV7, non-vaccine-related serotypes, such as 19A, circulated among pediatric groups. This serotype was reported to be multidrug resistant due to PCV7 implementation and antibiotic selection pressure. The high prevalence of CC271 clones was consistent with the high penicillin non-susceptibility rate found in IPD isolates pooled in this study.

According to a multicenter study conducted in China, ST320, ST271, and ST876 were the prevalent types among IPD isolates collected from children [52]. Another study showed that the CC876 prevalence increased from 0% in 1997–2000 to 96.4% in 2010–2012 in China, and the most important findings of this study were that this clone had high non-susceptibility rates to β-lactam antibiotics [53].

CC81 comprises ST81 (Spain^23F^-1, 5.2%, 19/365), and CC90 comprises ST90 (Spain^6B^-2, 4.1%, 15/365), which were also found at lower frequencies in this study. These complexes are listed as internationally spread resistant CCs [31].

## Conclusions

This is the first study to pool data for invasive pneumococcal diseases among children. The study has several limitations. First, the studies pooled were highly heterogeneous. Second, the studies included did not report the specific breakpoint from CLSI, and therefore we could not estimate the actual resistance of meningeal isolates. Long-term monitoring is required to estimate antibiotic resistance and serotype distributions in an effort to provide a policy to introduce a new PCV vaccine for Chinese children.

## Supplementary information

**Additional file 1.** PRISMA 2009 checklist.

## Data Availability

The data and materials is list as appendix.

## Abbreviations

CC: Clonal complexes
CIs: Confidence intervals
CLSI: *Clinical and Laboratory Standards Institute*
CNKI: China National Knowledge Infrastructure
EPI: Expanded Program on Immunization
HR: Hazard ratio
IPD: Invasive pneumococcal disease
MLST: Multilocus sequence typing
PCV: Pneumococcal conjugate vaccine
PNSP: Penicillin-non-susceptible pneumococci
*S. pneumonia*: *Streptococcus pneumonia*
STs: Sequence types
